# Supplementary material for: Effect of using home-based dynamic intermittent pneumatic compression therapy during periods of physical activity on functional and vascular health outcomes in chronic stroke: A randomized controlled clinical trial
Source: PLoS One. 2025 Feb 18;20(2):e0318942. doi: 10.1371/journal.pone.0318942 (PMC11835336; doi:10.1371/journal.pone.0318942)
Supplement: S1 Table — Data reported as mean ( ± SD) or as a percentage (%). S2 Table. Mean ( ± SD) vascular outcomes from IPC (n = 15) and CON (n = 16) groups at pre- and post-intervention. (DOCX) [file pone.0318942.s001.docx]

**Supplementary Tables**

**S1 Table.** Functional outcomes and SF-12 (physical and mental component) scores for IPC (n = 15) and CON (n = 16) groups at pre- and post-intervention. Data reported as mean (± SD) or as a percentage (%)

| **Outcome** | **Condition** | **Pre** | **Post** |
| --- | --- | --- | --- |
| **6MWT (m)** | IPC | 160 ± 69 | 180 ± 81 |
|  | CON | 170 ± 109 | 174 ± 117 |
| **RPE*** | IPC | 11.0 ± 3.3 | 11.4 ± 2.5 |
|  | CON | 12.2 ± 2.7 | 11.9 ± 2.0 |
| **TUG (s)** | IPC | 23.00 ± 8.92 | 22.72 ± 9.43 |
|  | CON | 29.08 ± 21.35 | 28.84 ± 24.25 |
| **10m walk (m/s)** | IPC | 0.61 ± 0.25 | 0.66 ± 0.33 |
|  | CON | 0.73 ± 0.51 | 0.72 ± 0.38 |
| **Sit-to-Stand (s)** | IPC | 23.96 ± 16.1 | 24.04 ± 17.09 |
|  | CON | 26.45 ± 21.30 | 26.68 ± 18.96 |
| **Fugl-Meyer (Upper)** | IPC | 30.3 ± 21.1 | 31.0 ± 21.7 |
|  | Control | 35.5 ± 20.5 | 36.8 ± 19.3 |
| **Fugl-Meyer (Lower)** | IPC | 22.9 ± 5.9 | 25.0 ± 4.1 |
|  | Control | 20.5 ± 9.6 | 21.9 ± 10.0 |
| **Berg Balance Scale** | IPC | 47.7 ± 3.53 | 46.9 ± 8.3 |
|  | Control | 41.2 ± 14.8 | 34.2 ± 18.8 |
| **ABC Scale (%)** | IPC | 62 ± 9 | 70 ± 12 |
|  | Control | 53 ± 21 | 60 ± 22 |
| **SF-12, PCS (%)** | IPC | 33.1 ± 7.8 | 33.7 ± 5.8 |
|  | Control | 29.5 ± 6.5 | 32.3 ± 7.7 |
| **SF12, MCS (%)** | IPC | 51.1 ± 6.7 | 50.1 ± 9.0 |
|  | Control | 52.0 ± 9.8 | 51.5 ± 9.0 |

*Note:* ABC, Activities, Balance Confidence scale; CON, Control; IPC, Intermittent pneumatic compression; MCS, SF-12 mental component score; PCS, SF-12 physical component score; RPE, Ratings of Perceived Exertion; TUG, Timed up-and-go test; 6MWT, Six minute walk test.

*RPE relates to the terminal value reported on completion of the 6MWT

**S2 Table.** Mean (± SD) vascular outcomes from IPC (n = 15) and CON (n = 16) groups at pre- and post-intervention.

| Outcome | Condition | Pre | Post |
| --- | --- | --- | --- |
| cfPWV (m/s) | IPC | 8.67 ± 2.14 | 8.38 ± 2.43 |
|  | CON | 8.70 ± 1.99 | 8.73 ± 1.94 |
| SBP (mmHg) | IPC | 147.4 ± 18.1 | 139.5 ± 15.6 |
|  | CON | 139.1 ± 17.5 | 138.9 ± 17.2 |
| DBP (mmHg) | IPC | 86.0 ± 9.3 | 82.2 ± 8.0 |
|  | CON | 83.3 ± 7.4 | 78.7 ± 11.8 |
| PP (mmHg) | IPC | 60.4 ± 17.9 | 58.1 ± 14.9 |
|  | CON | 55.9 ± 20.3 | 59.0 ± 20.0 |
| cSBP (mmHg) | IPC | 134.1 ± 14.0 | 127.1 ± 13.0 |
|  | CON | 127.9 ± 16.1 | 127.9 ± 15.7 |
| cDBP (mmHg) | IPC | 86.7 ± 9.1 | 83.2 ± 8.0 |
|  | CON | 84.3 ± 7.5 | 82.0 ± 8.6 |
| AIx (%) | IPC | 29.5 ± 8.0 | 29.4 ± 11.2 |
|  | CON | 31.4 ± 11.6 | 29.9 ± 6.6 |
| AIx75 (%) | IPC | 25.0 ± 9.7 | 26.0 ± 12.3 |
|  | CON | 26.6 ± 12.3 | 24.3 ± 11.5 |
| MAP (mmHg) | IPC | 105 ± 11 | 100 ± 10 |
|  | CON | 101 ± 9 | 99 ± 9 |
| HR (bpm) | IPC | 65.9 ± 8.9 | 67.2 ± 8.9 |
|  | CON | 66.4 ± 10.0 | 66.9 ± 13.5 |

*Note:* AIx, Augmentation index; AIx75, Augmentation index @ 75 bpm; cDBP, Central diastolic blood pressure; cfPWV, Carotid-femoral pulse wave analysis; CON, Control; cSBP, Central systolic blood pressure; DBP, peripheral diastolic blood pressure; IPC, Intermittent pneumatic compression; HR, Heart rate; MAP, Mean arterial pressure; PP, Pulse pressure; SBP, peripheral systolic blood pressure
