# Supplementary material for: Effect of using home-based dynamic intermittent pneumatic compression therapy during periods of physical activity on functional and vascular health outcomes in chronic stroke: A randomized controlled clinical trial
Source: PLoS One. 2025 Feb 18;20(2):e0318942. doi: 10.1371/journal.pone.0318942 (PMC11835336; doi:10.1371/journal.pone.0318942)
Supplement: S1 Appendix — (DOCX) [file pone.0318942.s002.docx]

**Appendix C_Research Aim and Overview**

***Background/Rationale***

Stroke is a leading cause of adult disability in the UK, with over 1.2 million people living with the debilitating effects (Stroke Association, 2020). It is estimated that approximately three quarters of individuals with stroke have fallen in the six months following discharge from hospital, and more recent research suggests that falls rates remained as high as 50% amongst those living in the community following stroke. With falls costing the National Health Service approximately £1.7 billion per year, neurorehabilitation is vital in order to improve balance and prevent falls in the stroke population (NHS Long-term plan, 2019). Evidence suggests that a key component of improving balance and mobility is through task-specific training that incorporates actual walking practice and lower limb muscle strengthening.

Dynamic pressotherapy is defined as a movement of compression/decompression, associated with a physical effort at variable intensity, in order to stimulate venous return. The GMOVE Suit (CE Marked; [GMOVE-SUIT - GMOVE SUIT](https://www.gmovesuit.com/en/); Appendix B) is a lower limb active pressotherapy device that may increase muscle activity and muscle tone, and support mobility renewal by stimulating the activation of lower-limb muscle pumping and muscle restraint during physical activity (via periods of compression/decompression of the device). In clinical populations, case studies with people with Ehlers-Danlos Syndrome, Parkinson’s Disease, Spinal Cord Injury, Stroke and Multiple Sclerosis, have demonstrated improvements in proprioception, functional outcomes, including the ‘Get Up and Go’ test, knee stability and control, and walking with greater hip and knee extension following regular usage of the GMOVE Suit.

Due to the portability of the device, there is substantial potential to use the GMOVE Suit in a home-based rehabilitation environment, whereby participants would have the potential to engage in daily physical activity whilst wearing the device. As the GMOVE Suit may encourage an increase in physical activity, there could be substantial benefit in stroke patients engaging in a structured (as determined by a neuro-physiotherapist), home-based rehabilitation programme which may improve vascular health (e.g., blood pressure), functional outcomes (walking speed, walking endurance), balance and general quality of life.

**Purpose**

The purpose of this research is to undertake a pilot randomised controlled study which will examine the effect of a home-based dynamic pressotherapy rehabilitation intervention, using the GMOVE Suit, on functional, vascular and quality of life parameters in individuals with chronic stroke.

**Objectives**

The objectives of this study are to:

1. Assess **feasibility** and **patient adherence** to using dynamic pressotherapy during home-based rehabilitation
2. Assess the effect of using dynamic pressotherapy on **clinical functional outcomes** of stroke patients
3. Assess the effect of using dynamic pressotherapy on **balance and mobility** of stroke patients
4. Assess the effect of using the dynamic pressotherapy on **vascular outcomes** (blood pressure, arterial stiffness) of stroke patients
5. Assess the effect of using dynamic pressotherapy on **quality of life** of stroke patients

***Methods***

Based on the findings of Ivey et al. and when using a two-tailed 5% significance level and a power of 80%, a sample size of 15 per group was calculated to detect a mean difference of 32m (pooled SD; 45m) for the 6MWT between groups. As such, and to account for potential participant drop-out, we will aim to recruit and randomise forty stroke patients to either a dynamic pressotherapy (GMOVE Suit) group (n=20) or a usual care control group (n = 20). The inclusion/exclusion criteria are as follows:

**Inclusion criteria:**

- Patients with clinical diagnosis of stroke (within 3 months and 5 years of diagnosis)
- Community patients that are medically stable
- Individuals who are able to stand and step (at least 10m) with (or without) an aid or assistance
- Cognitively aware of task demands

**Exclusion criteria**

- Unresolved deep vein thrombosis
- Unstable cardiovascular conditions
- Open wounds
- Active drug resistant infection
- Recent fractures of involved limb
- Peripheral arterial disease
- Severe osteoporosis
- Non weight bearing
- Diabetes

***Research Design***

The baseline and post-intervention (PI) assessments will be conducted at the University of Winchester and Hobbs Rehabilitation (dependent on outcome measures; see below). The rehabilitation training intervention will take place in participants’ home.

**Baseline assessment**

The initial baseline assessment will take place at the University of Winchester following a minimum 3 hour fast between the hours of 9.00 and 12.00pm. This part to the assessment is anticipated to take 75 minutes. On completion of a health history questionnaire (Appendix D), the following outcome measures will be collected.

*Demographics*

Height (m) and body mass (kg), Body Mass Index (kg·m^2^) will be measured. Height will be measured using a stadiometer while weight will be assessed using electronic scales.

*Vascular outcomes*

Following 15 minutes of quiet, supine rest, vascular outcomes will be assessed non-invasively by Pulse Wave Analysis (PWA) and Pulse Wave Velocity using the Vicorder (Skidmore Medical Ltd, Bristol, UK). To collect PWA measurements a cuff will be placed around the left arm of the participants. Initially, the cuff will inflate and peripheral blood pressure will be obtained. Following this a second inflation of the cuff will occur and PWA (an indirect indication of central aortic blood pressure) will be recorded. This will be repeated three times. Whilst supine, upper and lower body PWV will also be collected. PWV provides an indication of the speed that blood flows through the body, providing an indication of vascular health.

For PWV, cuffs will be placed around the neck**, upper thigh and ankle to collect carotid-femoral PWV (cfPWV) and femoral-ankle PWV (faPWV) data. For the cfPWV measurement, the distance between the neck cuff and the sternal notch and sternal notch to the upper thigh cuff will be measure. For the faPWV measurement, the distance between the cuff on the upper thigh and ankle will be measured. Following this, the neck cuff and the thigh cuff (cfPWV) will inflate simultaneously and then the thigh and ankle cuff will inflate simultaneously (faPWV), with both measures being repeated three times.

*** Note: The neck cuff includes a 1-cm cuff placed around the neck to detect the right carotid pulse. The neck cuff is designed in a way that it is placed over one carotid artery to avoid compression of the trachea and both carotid arteries at the same time. The cuff is automatically inflated simultaneously to 65 mm Hg (a mild pressure which is lower than diastolic blood pressure and therefore does not restrict bloodflow or affect breathing) and pulse waveforms are recorded for 3–5 s. This will be repeated three times with a one minute break in-between each measurement.*

*Functional Assessments*

Functional assessments will include the timed-up-and -go, 6-minute walk test and 10m walk test. These tests are commonly used functional outcome assessments with stroke patients (Wright et al. 2020). The timed-up-and-go will be completed three times with approximately a 2-minute recovery between tests to ensure adequate recovery. The 10m walk test will be performed three times and the fastest time will be used in the analysis.

*Questionnaires*

Participants’ quality of life will be assessed using the SF-12. The SF-12 (see Appendix F) has eight subscales: physical functioning, role limitations resulting from physical health issues, bodily pain, general health perceptions, vitality, social functioning, role limitations resulting from emotional problems, and mental health. From here, a physical and mental component score can be calculated.

*Habitual physical activity*

The IPAQ-SF will be used to capture physical activity at baseline and post-intervention. Participants will provide an indication of the amount of days and time they engage in vigorous, moderate and walking activity, as well as an indication of the amount of time they spend sitting each day.

*Physio-specific outcomes*

Seven days after the University assessment, participants will be assessed at Hobbs rehabilitation using some physio-specific measures. These measures will be undertaken by specialist neurophysiotherapists (Louis Martinelli and Amy Dennis-Jones), with support from the University research assistant, and is anticipated that it will take 45 minutes per participant. Assessments will include:

- *Functional Ambulation Classification:*
- *Sit-To-Stand (STS) symmetry:* Using the Tyromotion tymo force plate we can get patients to sit to stand off a standard surface (physio plinth) and monitor the force going through the right and left foot.
- *Fugl-Meyer: Assesses* motor recovery through a series of everyday tasks. It is widely used clinically to determine stroke severity and measure motor recovery. The assessment is comprised of 5 domains: motor function, sensation, balance, joint range and pain. Upper and lower body function will be reported.
- *BERG balance: Assesses* a patients ability to safely balance while performing a series of pre-determined tasks. It is comprised of 14 items. Scoring is based on a 5 point scale based on performance.
- *Activities Balance Confidence Scale:* This is a 15 item questionnaire whereby patients rate their perception of balance confidence across different tasks on a scale of 0 (no confidence) to 100 (highly confident). The scores are summed and the average score is reported.

**Randomisation and training programme**

On completion of the physio-specific measures participants will be randomised to either a dynamic pressotherapy group or to a usual care control using a computer-generated randomisation sequence ([www.randomizer.org](http://www.randomizer.org)). Those participants randomised to the pressotherapy group will be familiarised with the GMOVE Suit and will trained how to use it by the physiotherapists. The rehabilitation programme will include activities such as walking, stepping, squats etc. Thereafter, participants will use the GMOVE Suit at home for 12 weeks. Every other week (week 1, 3, 5, 7, 9, 11) they will receive feedback from the physiotherapists (either face-to-face or online) with regards to the necessary settings to encourage physical progression. These sessions are anticipated to take between 15 and 30 minutes each.

**Post-intervention (PI) assessment:**

The PI assessment will be identical to the baseline assessment

**Data analysis**

Quantitative data will be collected throughout the baseline and post-intervention assessments through various laboratory-based and physio-specific measures. Participant details will be anonymised, outcome variables will be coded where necessary (e.g., randomised groups) and entered into SPSS (v26) and analysed using appropriate parametric or non-parametric testing procedures pending upon the data distribution. Independent samples t-tests (i.e., age, time since stroke, FAC) or chi-square tests (e.g., sex) will be used to compare demographic characteristics and clinical outcomes (e.g., PWA, cfPWV, 6MWT) between groups at baseline. For the main analyses , differences in baseline data will be considered and reported as a covariate (e.g., if using ANCOVA). Effect sizes will be reported where necessary and alpha will be set at 0.05

Daily and average wear time of using the GMOVE suit will be recorded throughout the 12 week programme for all participants. Daily RPEs will also be recorded.
