# Supplementary material for: Effect of using home-based dynamic intermittent pneumatic compression therapy during periods of physical activity on functional and vascular health outcomes in chronic stroke: A randomized controlled clinical trial
Source: PLoS One. 2025 Feb 18;20(2):e0318942. doi: 10.1371/journal.pone.0318942 (PMC11835336; doi:10.1371/journal.pone.0318942)
Supplement: Attachment — Submitted filename: Response to Reviewers.docx [file pone.0318942.s005.docx]

PONE-D-24-13580R1

Effect of using home-based dynamic intermittent pneumatic compression therapy during habitual physical activity on functional and vascular health outcomes in chronic stroke: A randomized controlled clinical trial

Please include the following items when submitting your revised manuscript:

- A rebuttal letter that responds to each point raised by the academic editor and reviewer(s). You should upload this letter as a separate file labeled 'Response to Reviewers'.
- A marked-up copy of your manuscript that highlights changes made to the original version. You should upload this as a separate file labeled 'Revised Manuscript with Track Changes'.
- An unmarked version of your revised paper without tracked changes. You should upload this as a separate file labeled 'Manuscript'.

6. Review Comments to the Author

**Reviewer #1: Although the authors have addressed most of the points generated during the review, please
• Refine the abstract to better highlight the broader clinical implications of IPC therapy for stroke rehabilitation.**

We have adjusted the conclusion of the abstract to better reflect the broader clinical implications as advised. It now reads:

*‘The observed improvements in functional mobility, cardiovascular health, increased physical activity and reduced sedentary time demonstrates important clinical implications of ‘home-based’ IPC therapy as a clinical training aid for stroke rehabilitation. Home-based IPC therapy could serve as an adjunct to conventional rehabilitation, however, further research is needed to determine whether IPC therapy can sustain or improve function over time for individuals in the chronic stage of recovery.’*

**• Provide additional detail on the randomisation process (clearer explanation of the block sizes and strata) and handling of missing data in the methodology.**

To improve the clarity the following information has been adapted regarding information on the Block randomization process:

*‘Block randomization was employed to ensure an equal distribution of participants across the groups. The study used eight blocks, each consisting of four participants, two assigned to the IPC group and two to the Control group, to meet the required sample size. Within each block, participants were randomized to either the IPC or control group.’*

In terms of the handling of missing data, we are unsure what further information is required as we feel that the information provided clearly articulates the process. On page 14 under the Data analysis subheading we stated:

*‘An intention-to-treat analysis was applied to all repeated-measures procedures, with participants analyzed as randomized, using the last available data to replace any subsequent missing assessments’*

**• Clarify the integration of IPC with habitual physical activity to avoid ambiguity in the intervention description and explain how this integration differs from traditional exercise interventions.**

Thank you for your comments. We have removed the term “habitual” from the manuscript when referring to the IPC intervention, as it does not accurately reflect the prescribed program. Participants were instructed to wear the IPC device for at least 30 minutes daily, during which they were encouraged to walk and engage in activities such as step-ups and sit-to-stands at prescribed RPEs. While the program shares some similarities with a traditional exercise program, its intensity is likely lower than that of a gym-based aerobic or resistance training program, due to the focus on walking and the specific needs of the population group.

**• Expand the discussion of non-significant findings and provide hypotheses for why certain outcomes did not improve.**

We have provided additional context as to why changes in the TUG were not observed. We have adapted the second paragraph to the discussion to now read:

*‘The TUG test primarily evaluates mobility, balance and the functional movements of standing-up, walking a short distance, turning and sitting down. Although the IPC intervention included movements that overlap with the tasks assessed in the TUG test, the primary focus was on using the IPC device during regular walking activities. It did not specifically target balance or the complex movement transitions, such as standing up, turning, and sitting down, that are key components of the TUG test. This may explain why no significant improvements were observed in the TUG test’.*

However, when discussing the Fugl Meyer and BBS results, we do not think any further information is needed due to the following:

*‘…, there were no significant changes in BBS or Fugl Meyer (upper and lower-extremity scores) for either condition. This may again relate to the fact that walking was the primary exercise activity in the intervention, with balance-type activities of secondary importance.’*

Finally, when commenting on the non-significant findings related to cfPWV and AIX, the explanation around higher-intensity exercise has been expanded to now read:

*‘As a 1 m/s reduction in PWV is the minimal clinically important difference and is strongly associated with decreased CVD risk [48], our lower-intensity IPC program may not be as effective in impacting upon regional arterial stiffness. Since AIx is a derivative of PWV, it is unsurprising that there were no differences between groups for such outcomes. Previous research has consistently demonstrated favorable changes in vascular outcomes (e.g., cfPWV) when moderate to vigorous volumes of physical activity are prescribed in training interventions [49, 50]. Higher-intensity exercise increases shear stress on the arterial walls, stimulating the release of nitric oxide from the endothelium. This promotes vasodilation, improves arterial compliance, and reduces arterial stiffness, as reflected in a decrease in PWV [49]. Lower-intensity exercise, such as our IPC intervention, may not initiate such physiological adaptations. However, they may promote better adherence and be more attainable, as enjoyment and well-being are strong motivators for long-term engagement [51] [52].’*

• **Acknowledge the potential selection bias in more detail, particularly the limitations of recruiting highly motivated participants from a private clinic. Discuss how this may affect the external validity of the findings.**

We have restructured this section to the manuscript so that the limitations associated with the sample are clearly articulated. It now reads:

*‘Firstly, there was evidence of sampling bias, as the recruited sample may not represent a typical stroke population. Participants were recruited from a private neuro-physiotherapy practice, meaning they were likely highly motivated to participate in rehabilitation due to the financial investment associated with private physiotherapy services. This ultimately limits the generalizability of the study findings. Additionally, the study's external validity is affected because highly motivated individuals are more likely to fully engage with and adhere to study protocols. This higher level of compliance could make the intervention seem more effective than it actually would be in a broader stroke population, potentially resulting in an overestimation of its true efficacy.’*

**• Specify future research directions more clearly, by providing more concrete suggestions for future studies, such as exploring different stroke severity levels or testing IPC therapy in combination with more structured exercise regimens.**

We have revised the future research section to provide a clearer outline of potential directions for future work. It now reads:

*‘If the latter, further research is needed to determine whether alternative parameters (e.g., heart rate) could be a better tool to progress participants, whether varying pressures and IPC device dosage elicit more favorable effects on patients, and whether the technology is more suited for acute or sub-acute stroke patients, or bilateral populations (e.g., multiple sclerosis, spinal cord injury). Considering the sampling bias identified in this study, future research should also evaluate the generalizability of the observed positive effects of IPC therapy to a broader stroke population. Furthermore, investigations could examine whether stratification by stroke severity (e.g., minor, moderate, severe) yields greater improvements in functional mobility and cardiovascular health, while additional studies are warranted to assess the potential synergistic effects of integrating IPC therapy with structured exercise interventions, e.g., 8- to 12-week aerobic training program.’*

**Reviewer #2: (No Response)**

**Reviewer #3: The Authors have revised rather extensively their manuscript according to my comments and those by the other Reviewers. I have no further comments.**

Thank you for reading the revised manuscript and for your engagement in the process.
